# Removal of Pharmaceutical and Personal Care Products (PPCPs) from Municipal Waste Water with Integrated Membrane Systems, MBR-RO/NF

**DOI:** 10.3390/ijerph15020269

**Published:** 2018-02-05

**Authors:** Yonggang Wang, Xu Wang, Mingwei Li, Jing Dong, Changhong Sun, Guanyi Chen

**Affiliations:** 1 School of Environmental Science and Engineering, Tianjin University, No. 92, Weijin Road, Nankai District, Tianjin 300072, China; wangyonggang@cee.cn (Y.W.); chen@tju.edu.cn (G.C.); 2 Beijing Municipal Research Academy of Environmental Protection, No. 59, Beiyingfang Middle Street, Xicheng District, Beijing 100037, China; limingwei@cee.cn (M.L.); dongjing@cee.cn (J.D.) ; sunchanghong@cee.cn (C.S.); 3 National Engineering Research Center for Urban Environmental Pollution Control, No. 59, Beiyingfang Middle Street, Xicheng District, Beijing 100037, China

**Keywords:** PPCPs, wastewater, membrane bioreactor, reverse osmosis, nanofiltration, removal mechanisms

## Abstract

This study focuses on the application of combining membrane bioreactor (MBR) treatment with reverse osmosis (RO) or nanofiltration (NF) membrane treatment for removal of pharmaceuticals and personal care products (PPCPs) in municipal wastewater. Twenty-seven PPCPs were measured in real influent with lowest average concentration being trimethoprim (7.12 ng/L) and the highest being caffeine (18.4 ng/L). The results suggest that the MBR system effectively removes the PPCPs with an efficiency of between 41.08% and 95.41%, and that the integrated membrane systems, MBR-RO/NF, can achieve even higher removal rates of above 95% for most of them. The results also suggest that, due to the differences in removal mechanisms of NF/RO membrane, differences of removal rates exist. In this study, the combination of MBR-NF resulted in the removal of 13 compounds to below detection limits and MBR-RO achieved even better results with removal of 20 compounds to below detection limits.

## 1. Introduction

Pharmaceuticals and personal care products (PPCPs) are a class of emerging contaminants, which include commonly used medicinal, cosmetic and personal hygiene products. PPCPs have been widely detected in surface, ground, coastal and even drinking water [1,2,3,4]. Great concerns have been raised about PPCPs due to their potential adverse impacts on the ecological system and human health.

PPCPs contain a large and diverse group of organic compounds, including pharmaceutically active compounds, endocrine disrupting compounds and so on. This kind of compound has a potential physiological effect on the water environment, water ecology and human health. For example, it can cause fish’s nephridial tissue necrosis, influence the growth of alga and duckweed, enhance the microbial resistance to antibiotics, etc. [5,6,7]. Compared with common pollutants, this compound has strong polarity, low mass concentration and biological accumulation feature. In addition, it exists in bodies of water with very low concentration (<1 μg/L), which makes it quite difficult for urban sewage treatment plants to remove it [8]. Therefore, PPCPs have a huge potential ecological risk for both aquatic organisms and humans, and an efficient treatment process has become the focus of many scholars from home and abroad.

Many studies have explored different treatment methods to remove PPCPs from wastewater and receiving waters, including conventional activated-sludge, soil aquifer treatment, advanced oxidation process and biomembrane process [9,10,11,12,13,14,15]. Among them, biomembrane process is one of the most effective treatment processes. With the development of such technology, it has become more efficient, convenient and economically viable, and the membrane processing technique now plays an increasingly important role in sewage treatment. According to Jelena et al. [16] membrane bio-reactor (MBR) has a better effect (>80%) on most pharmaceuticals (naproxen 99.3%, ofloxacin 94.0%, bezafibrate 95.8%, and paroxetine 89.7%) than conventional activated-sludge. Katsuki et al. [17] have studied reverse osmosis (RO) membrane’s effect on 11 endocrine disrupting chemicals (EDCs) and pharmaceutically active compounds (PhACs), and it shows that the polyamide membrane has achieved a good removal effect on 2-naphthol, 4-phenylphenol, caffeine, bisphenol A, sulfamethoxazole and 17β-Estradiol with rate of 51% to 91%. Boleda et al. [18] and Sahar et al. [19] have proven that ultrafiltration (UF) - RO double membrane process has good removal efficiency (90% on average) on PhACs and EDCs. Dolar et al. [20] focus on the study of MBR-RO process’s efficiency on twenty multiple-class pharmaceuticals (including psychiatric drugs, macrolide antibiotics, β-blockers, vsulfonamide and fluoroquinolone antibiotics), and treatment has exhibited excellent overall removal of selected emerging contaminants with removal rates above 99%. Establishing MBR-NF(nanofiltration) treatment process, Alturki et al. [21] have studied different nanofiltration membranes’ efficiency to forty trace organics, including pharmaceutically active compounds, steroid hormones, industrial compounds and pesticides, and the removal rates are all above 95%.

Thus, the main goal of this work is to assess the removal efficiency (after each step and overall) of selected contaminants (27 PPCPs) from typical municipal wastewater by using an integrated membrane system (MBR-RO and MBR-NF) on a pilot scale. 

## 2. Materials and Methods

### 2.1. MBR-RO/NF Pilot Plant

**Figure 1:**
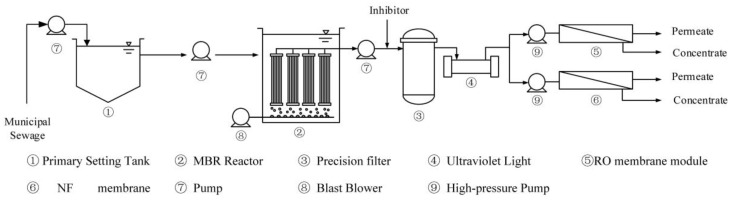
Schematic representation of the MBR-RO/NF pilot plant showing different compartments, flow directions, main instruments and equipment.

A pilot scale MBR-RO/NF system was employed in this study (Figure 1). The MBR system consisted of a stainless-steel reactor with an active volume of 9 L, one air pump, a pressure sensor, and influent and effluent pumps. Forty-eight membrane microfiltration (MF) membrane components supplied by PEIER (Jiangsu, China) were used in this apparatus (Table 1). This membrane had a nominal pore size of 0.08–0.3 μm. Each module had an effective membrane surface area of 0.034 m^2^. An electrical magnetic air pump (seko, Milano, Italy) with a maximum air flow rate of 630 L/min was used to aerate the MBR system and reduce fouling and formation of cake via a diffuser located at the bottom of the reactor. Transmembrane pressure was continuously monitored using a high-resolution pressure sensor which was connected to a personal computer for data recording purposes.

The temperature of the reactor was kept constant using a chiller/heater (Thermo NESLAB RTE-7, Bacchus Marsh, Australia), equipped with a stainless-steel heat exchanging coil. The temperature inside the reactor was 16.7 ± 0.5 °C during the entire sampling campaign. The flow rate of the influent pump was matched to that of the permeate pump to maintain a constant reactor volume. The MBR was operating with a hydraulic retention time (HRT) of 3.2 h, average pH value of 7.8. The solid retention time (SRT) was 40 days and the production flow was 0.8 m^3^/h during the sampling campaign. 

The precision filter (Ф195 × 500, Tongzhou Zhiyuan, Beijing, China) and ultraviolet light (NLC-25/65, Tongzhou Zhiyuan, Beijing, China) were applied to prevent the NF/RO membrane from fouling by particles and bacteria, respectively. Research shows that ultraviolet (UV) alone (without NF or RO membrane) was not effective in reducing the PPCPs concentration [22,23,24].

The reverse osmosis and nanofiltration membrane (Duraslik^TM^ , GE, California, USA) were used in this study, which had an effective membrane area of 2.5 m^2^ and 2.2 m^2^, respectively. The RO unit was equipped with a high-pressure pump (CDL2-11/1.1 KW, Nanfang Pump Industry, Suzhou, China) capable of providing pressures up to 1.32 MPa and a flow rate of 13 L/min. The NF unit was equipped with high-pressure pump (CDL2-5/0.55 KW, Nanfang Pump Industry, Suzhou, China) capable of providing pressures up to 0.7 MPa and flow rate of 13 L/min.

Permeate flow was measured by a digital flow meter (Sierra, Shanghai, China) connected to a PC, and the cross-flow rate was monitored using a rotameter.

### 2.2. Model Contaminants

The 27 kinds of PPCPs selected in the study are commonly found in wastewater and natural waters (see Table 2). The selected contaminants include organic compounds with molecular weights in the range between 194.19 g/mol (caffeine) and 837.05 g/mol (roxithromycin). The intrinsic hydrophobicities of these compounds vary significantly, as is also reflected by their octanol-water partitioning coefficient (Log*Kow*) values.

### 2.3. Sample Collection and Analysis

Samples were taken during a one-week period and both influent and effluent sample were collected and analyzed for PPCP concentration. The sampling points were: (1) municipal wastewater-sewer (influent); (2) MBR effluent; and (3) permeate of RO/NF element. Water samples were collected in 1-L amber glass bottles. To ensure the stability of the target, the pH of the samples was adjusted to 3 with H_2_SO_4_ (40%). Samples were kept at 4 °C during preparation (1 h) and were vacuum filtered through 1.0 μm glass microfiber filter (Whatman, Maidstone, UK). 

The analysis of the target compounds was based on a previously developed method [28]. Analytes were extracted using 500 mg/6 mL hydrophilic/lipophilic balance (HLB) cartridges (Waters, Millford, MA, USA). Cartridges were pre-conditioned with 5 mL of methanol alcohol, 5 mL of amprolium HCl and 3 mL of reagent water. The sample was loaded onto the cartridges at 4–5 mL/min, after which the cartridges were rinsed with 5 mL of methanol alcohol aqueous solution (5%), 5 mL of reagent water and dried with a stream of nitrogen for 40 min. Analytes were eluted with 10 mL of methanol followed by 5 mL of dichloromethane/acetone (7/3, *v*/*v*) methanol/MTBE into centrifuge tubes. The resulting extracts were concentrated using vacuum assisted evaporation to approximately 100 μL. The extracts were brought to a final volume of 1 mL with methanol. Analytes were separated using an Agilent (Waters, Milford, MA, USA) 1290 series ultra performance liquid chromatography (UPLC) system equipped with an OAsis HLB column (Waters, Milliford, MA, USA). Mass spectrometry was performed using an Agilent 6420 triple quadrupole mass spectrometer (Waters, Milford, MA, USA) with positive electro-spray modes (ESI+) [28].

Considering that PPCPs have rather different physicochemical characteristics, their removal during treatment is expected to be diverse. The literature shows that the removal efficiency is generally computed as the percentage of reduction between the dissolved aqueous phase concentration of the contaminant in the influent and that in the effluent. Except for a few studies, concentrations in sludge or suspended solid are generally not considered or measured, likely because of the difficulty to sample and analyze such complex matrices [29].

## 3. Results and Discussion

### 3.1. PPCPs in the Influent

In Table 3, the range of levels observed in influent wastewaters for each contaminant (with their mean values) are presented. Levels of target compounds were in the ng/L range but concentrations of some of them exceeded 1 μg/L range (caffeine, metoprolol and azithromycin). The process received domestic (100%) wastewater and thus high concentrations of selected compounds were found.

Among the selected contaminates, the highest concentrations in influent were found for nervous stimulant caffeine (8.53–33.7 μg/L), β-blocker metoprolol (0.437–3.21 μg/L), macrolide antibiotics azithromycin (0.047–4.42 μg/L) and surfactant bisphenol A (up to 1.52 μg/L). Other authors [30,31,32] presented similar ranges (ng/L to μg/L) for the pharmaceuticals and personal care products found in the municipal wastewaters.

Caffeine was present in the highest concentration (33.7 μg/L) making it in agreement with concentrations presented by other authors [33,34]. The reason is that caffeine is widely distributed in coffee, tea and other beverages, and flow into sewage with residue [35]. Macrolide antibiotics are largely excreted into sewage in their unchanged forms at excretion rates greater than 60% and they are usually found in wastewater at high concentrations [30]. Relatively high concentrations of azithromycin (up to 4.42 μg/L) were observed. Despite its high consumption, other kinds of macrolide antibiotics (such as roxithromycin, clarithromycin, and erythromycin-H_2_O) were found at much lower levels (10 μg/L) than azithromycin. In addition, the same result has been found in other studies [36].

On the other hand, bisphenol-A was present in relatively high concentration (up to 1.5 μg/L), which is in agreement with concentrations presented by other authors [33]. Among many pollutants, estrone, 17β-Estradiol and estriol are the three estrogen compounds that gain the most attention in present studies. The concentration of 17β-Estradiol ranged 10.6–54.1 ng/L, while concentrations of the other two compounds are all at the 100 ng/L level, which is quite close to the concentration of estrogen compounds in Zhang’s [37] study.

### 3.2. Removal of PPCPs by MBR

**Figure 2:**
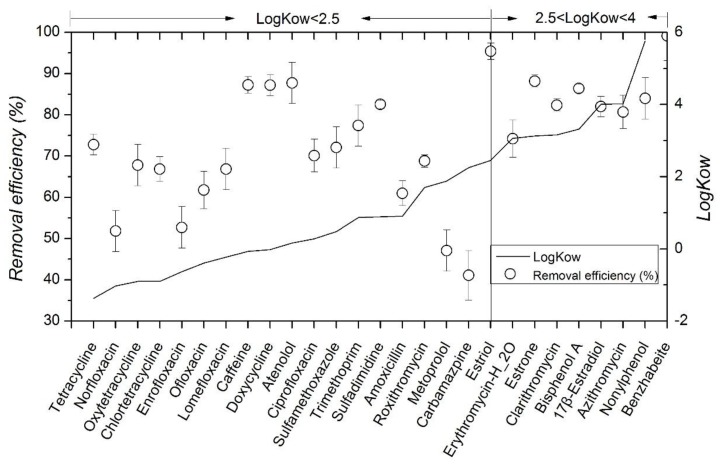
Removal efficiency of the model contaminants and their corresponding hydrophobicity (Log*Kow*) by MBR treatment.

MBR’s average removal efficiency to the selected contaminants is presented in Figure 2. MBR’s main removal pathways to organic compounds are biodegradation (biotic)/absorption (abiotic) and biotransformation. Adsorption includes membrane surface adsorption and its settled layer adsorption. Biotic and abiotic process could not easily be distinguished in this study and therefore removal efficiency refers to overall removal result under two mechanisms.

Given the diverse physicochemical properties of the 27 contaminants selected in this study, it is not surprising that their removal efficiency varied significantly. Lower removal is observed for carbamazpine (41%) and metoprolol (47%). In contrast, for several other compounds, including estriol (95%), benzhabeite (88%), caffeine (88%) and atenolol (87%), the removal efficiency is relatively high. This is in accordance with the research results of Alturki et al. [21].

For the macrolide antibiotics, MBR treatment shows mean removal efficiency from 74% to 82%. Under typical wastewater conditions, many macrolides can be adsorbed into biomass, mainly attributed to hydrophobic interactions due to their high Log*Kow* partitioning coefficients (Table 1). Many macrolides are positively charged whereas sludge surface is predominantly negatively charged, which leads to adsorption of these compounds to biomass via cation exchange processes [38]. 

Sulfonamides is partly removed (61.4%) with MBR, likely due to a moderate sorption to sludge and to its limited biodegradability. Chemicals with Log*Kow* < 2.5, as is the case with sulfamethoxazole and sulfadimidine, are considered to have low hydrophobic sorption potential. Göbel et al. [29] have described lower removal of sulfamethoxazole (37–38%) with a MBR system.

MBR’s removal efficiency rates on fluoroquinolones are 51.8% (norfloxacin), 70.1% (ciprofloxacin), 61.8% (ofloxacin), 66.9% (lomefloxacin) and 52.7% (enrofloxacin); its removal efficiency rates to tetracycline antibiotics are 67.80% (oxytetracycline), 72.79% (tetracycline), 66.8% (chlortetracycline) and 75.6% (doxycycline); and the Log*Kow* values are all below 2.5. Therefore, this kind of compound has relatively low adsorption potential energy and weak hydrophobic property, which brings difficulty to MLSS’s (mixed liquor suspended solid) adsorption and further biodegradation process. As a result, MBR has a low removal efficiency to the three organisms.

Removal of carbamazepine is relatively poor (47.23%). Poor elimination of this neutral has been reported by many authors [16,39,40,41]. Carbamazpine possesses very weak biodegradability under low concentration, thus it is hard for activated sludge to conduct effective biodegradation. Although carbamazpine could adsorb on the surface of activated sludge, it fails to be degraded effectively. Besides, the carbamazpine adsorbed from activated sludge would flow out with MBR water.

MBR’s removal efficiency to retardant drugs are 87.7% (atenolol) and 47.1% (metoprolol). These removal rates are the same as the study by Radjenovic et al. [16], and much lower than those observed by Dalar et al. [20]. Maurer et al. [42] found that removal mechanism of β-blockers with MBR sludge is mainly biodegradation, whereas only in the case of propanolol, sorption was a possible removal method. 

**Figure 3:**
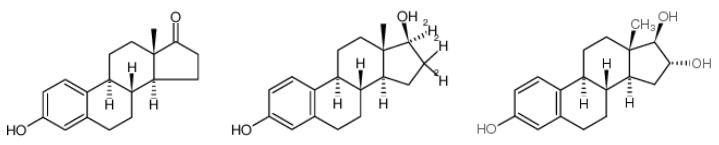
The chemical structure of Estrone/17β-Estradiol/Estriol.

MBR’s removal efficiency to three estrogens is 88.2% (estrone), 82% (17β-Estradiol) and 95.4% (Estriol), and their Log*Kow* values are 3.13, 4.01 and 2.45, so medium or high absorption potential energy is presented. According to the research, absorption would easily happen when OH hydroxy on benzene ring and C=O hydroxy on adsorbent form into hydrogen bond, and it is easier to remove [43]. Among the three estrogens, estriol has slightly lower Log*Kow* value, but there are three hydroxyls in its molecular structure (Figure 3). Then, Estriol forms into hydrogen bond with C=O hydroxy on mixed liquid suspended solids (MLSS), which strengthens adsorption, and a higher removal efficiency is achieved.

### 3.3. Removal of PPCPs by MBR-RO/NF System

**Figure 4:**
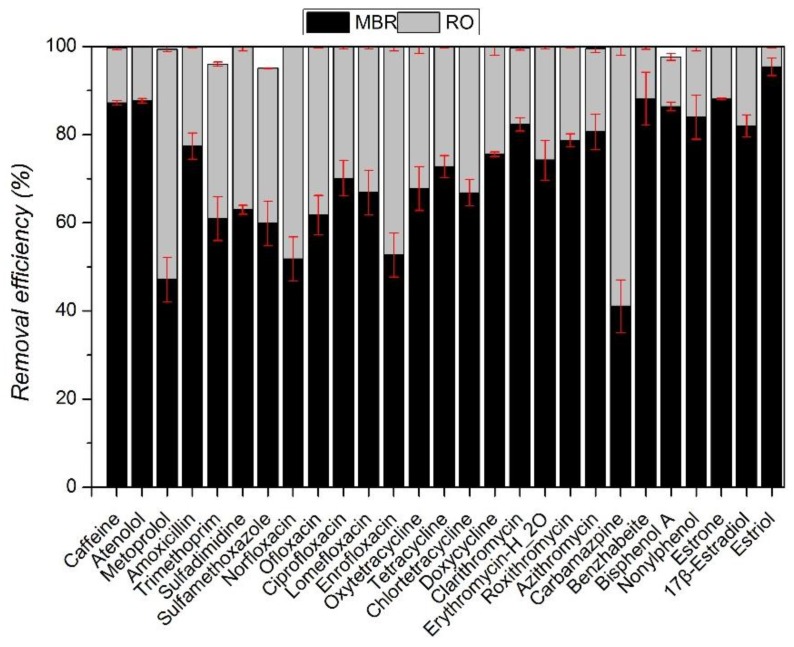
Overall removal efficiency of the selected contaminants by MBR-RO system.

Results reported in Figure 2 reconfirm the limitation of MBRs with respect to the removal of some hydrophilic and biologically persistent trace organic compounds. However, since most of these problematic compounds are hydrophilic, there is a potential for use of NF/RO membranes to more effectively remove them. The overall removal of the 27 selected contaminants with MBR-RO/NF is presented in Figure 4. RO/NF membranes can both complement MBR treatment very well, since the majority of compounds studied in the influent were almost entirely removed or concentrations were below the detection limit of the analytical technique after RO/NF treatment. Overall removal rates are greater than 95%, which means that additional removal of selected contaminants with RO and NF membrane is higher than 95% and are in agreement with results obtained by other researchers [21,31,44,45]. Snyder et al. [46] obtained removal rates of various pharmaceuticals (antibiotics, psychiatric control, anti-inflammatory, etc.) higher than 90%. Wang et al. [47] found that most of the 40 trace organic compounds studied were effectively rejected by NF membrane.

**Figure 5:**
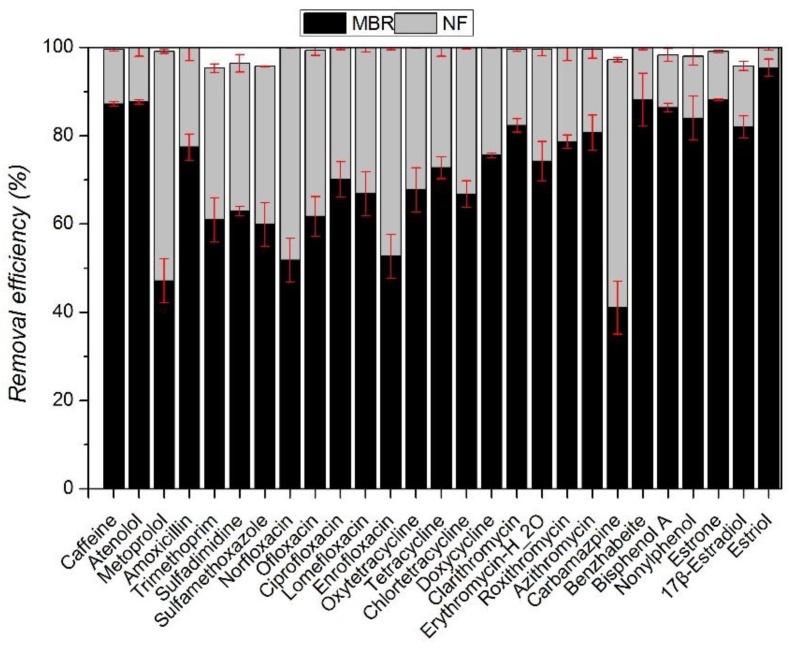
Overall removal efficiency of the selected contaminants by MBR-NF system.

Even though high rates of removal of most of the 27 compounds selected in this study were achieved with the combination of MBR-NF/RO treatment processes, there are also variations. The combination of MBR treatment and the NF membrane resulted in removal of 13 compounds to below detection limits (Figure 4). MBR and the RO membrane achieved even better results with removal of 20 compounds to below detection limits (Figure 5). Despite the significant variation in the concentrations of these compounds in the MBR effluent of up to 10 μg/L, their concentrations in the final RO permeate were only marginally above the analytical detection limits.

Removal of contaminants by RO is determined by complex interactions of electrostatic and other physical forces acting among the specific solute, the solution and the membrane itself. Main removal mechanisms in RO membranes are steric hindrance, electrostatic interaction and hydrophobic interaction between compounds and the membrane [17,40]. Considering the hydrophobicity, a compound that possesses strong hydrophobicity (Log*Kow* > 2.5) can attach to the membrane’s polymer matrix, and the possible removal mechanism may be hydrophobicity interaction. In contrast, electrostatic attraction or repulsion forces can influence the rejection of some contaminants in RO membrane due to their charge. Generally, RO membranes can obtain a better removal performance, due to aperture of the reverse osmosis membrane is less than 1 nm, which can able to intercept most of soluble organic matters [48,49]. Among various parameters affecting removal of PPCPs by NF, namely physicochemical properties of the PPCPs (charge characteristics, hydrophobicity and molecular weight) and membranes (molecular weight cut off and surface charge), the molecular weight cut off (MWCO) effect was found to be the most critical aspect. The molecular cut off of Duraslik^TM^ 2540 NF membrane is 300. For selected contaminants whose relative molecular weight is less than 300, such as sulfadimidine (278.33) and sulfamethoxazole (253.27), removal efficiency is lower, while, for pollutants whose molecular weight is more than 300, NF’s removal efficiency rate is above 99%.

## 4. Conclusions

The rejection performance of MBR-RO and NF process was tested with twenty-seven selected PPCPs from municipal wastewater in this study. The concentration variation of the selected contaminant in feed water was first monitored. Result showed that 27 PPCPs were in relatively high concentrations (caffeine, even up to 33.7 μg/L). Average concentrations of some PPCPs ranged from 7.1 ng/L (pyrimethamine antibiotic) and 75.9 ng/L (estrogen) to 468.5 ng/L (macrolide antibiotics). Removal efficiency of MBR varied significantly (41–95%) depending on compound, which was due to the diverse physicochemical properties of the 27 target compounds. High removal rates (to levels below LOQ) were achieved with the combination of MBR–RO/NF treatment for all compounds selected. Hydrophilic PPCPs compounds were effectively removed by NF/RO membranes selected in this study. Size exclusion and electrostatic attraction or repulsion were presumed to be the primary mechanisms involved in the removal of target compounds with RO membranes. Among various parameters affecting the removal of PPCPs by NF, the MWCO effect was found to be the most critical aspect.

## Figures and Tables

**Table 1:** Properties of three membrane modules.

Membrane Component	Texture	Type	Rejection (%) ^a^		Effective Area (m^2^)	General Operation Pressure (KPa)	General Operation Flux (LMH)
			Average	Minimum			
RO	PA ^b^	Duraslick RO 2540	98.6% (NaCl)	97% (NaCl)	2.5	1379	15–25
NF	PA ^b^	Duraslick NF-2540	98.6% (MgSO_4_)	96% (MgSO_4_)	2.2	690	15–25
MBR	PVDF ^c^ + PET ^d^	PEIER-B-80	-	-	0.8	-	-

^a^ taking test after running 24 h, the average rejection of single membrane might be −15% ~ +25%; ^b^ PA: Polyamide; ^c^ PVDF: Polyvinylidene fluoride; ^d^ PET: Polyethylene terephthalate.

**Table 2:** Physicochemical properties of the selected compounds.

Analytes	MW (g/mol)	Formula	CAS Number	Log*Kow*	pKa *	Solubility * (mg/L)	Classification
Caffeine	194.19	C_8_H_10_N_4_O_2_	58-08-2	−0.07	6.1; 0.73	2.16 × 10^4^	Stimulant
Atenolol	266.34	C_14_H_22_N_2_O_3_	29122-68-7	0.16	13.88; 9.16	N/A	β--blocker
Metoprolol	267.36	C_15_H_25_NO_3_	51384-51-1	1.88	9.68	N/A	β--blocker
Amoxicillin	365.4	C_16_H_19_N_3_O_5_S	26787-78-0	0.91	N/A	N/A	β-lactams Antibiotic
Trimethoprim	290.32	C_14_H_18_N_4_O_3_	738-70-5	0.87	6.3; 4.0; 7.2	12100	Pyrimethamine antibiotic
Sulfadimidine	278.33	C_12_H_14_N_4_O_2_S	57-68-1	0.89	7.4	1500	Sulfonamides antibiotics
Sulfamethoxazole	253.27	C_10_H_11_N_3_O_3_S	723-46-6	0.48	2.1; 5.81; 1.39	610	Sulfonamides antibiotics
Norfloxacin	319.33	C_16_H_18_FN_3_O_3_	70458-96-7	−1.03	N/A	N/A	Fluoroquinolone antibiotics
Ofloxacin	361.37	C_18_H_20_FN_3_O_4_	82419-36-1	−0.39	N/A	N/A	Fluoroquinolone antibiotics
Ciprofloxacin	331.34	C_17_H_18_FN_3_O_3_	85721-33-1	0.28	N/A	3.0 × 10^4^	Fluoroquinolone antibiotics
Lomefloxacin	351.35	C_17_H_19_F_2_N_3_O_3_	98079-51-7	−0.23	N/A	N/A	Fluoroquinolone antibiotics
Enrofloxacin	359.4	C_19_H_22_FN_3_O_3_	93106-60-6	−0.63	N/A	N/A	Fluoroquinolone antibiotics
Oxytetracycline	460.43	C_22_H_24_N_2_O_9_	79-57-2	−0.9	3.27	N/A	Tetracycline antibiotics
Tetracycline	444.44	C_22_H_24_N_2_O_8_	60-54-8	−1.37	3.30	N/A	Tetracycline antibiotics
Chlortetracycline	478.88	C_22_H_23_ClN_2_O_8_	57-62-5	−0.9	3.30	N/A	Tetracycline antibiotics
Doxycycline	444.44	C_22_H_24_N_2_O_8_	564-25-0	−0.02	3.30	N/A	Tetracycline antibiotics
Clarithromycin	747.95	C_38_H_69_NO_13_	81103-11-9	3.16	8.9	0.33	Macrolide antibiotics
Erythromycin-H_2_O	715.916	C_37_H_65_NO_12_	23893-13-2	3.06	8.9	1.44	Macrolide antibiotics
Roxithromycin	837.05	C_41_H_76_N_2_O_15_	80214-83-1	1.7	8.8	0.019	Macrolide antibiotics
Azithromycin	748.98	C_38_H_72_N_2_O_12_	83905-01-5	4.02	8.8	N/A	Macrolide antibiotics
Carbamazpine	236.27	C_15_H_12_N_2_O	298-46-4	2.25	13.90; −0.49	112	Antiepilepti
Benzhabeite	361.82	C_19_H_20_ClNO_4_	41859-67-0	N/A	N/A	N/A	Antihypercholesterolemic
Bisphenol A	228.29	C_15_H_16_O_2_	80-05-7	3.32	9.73	120	Plasticizer
Nonylphenol	220.35	C_15_H_24_O	25154-52-3	5.76	10.14	N/A	Plasticizer
Estrone	270.37	C_18_H_22_O_2_	53-16-7	3.13	10.25; 10.5	N/A	Hormone
17β-Estradiol	272.38	C_18_H_24_O_2_	50-28-2	4.01	10.27; 10.4	3.6	Hormone
Estriol	288.38	C_18_H_24_O_3_	50-27-1	2.45	10.25; >15	444	Hormone

* Physico-chemical information was obtained from [19,25,26,27]. N/A: Not applicable.

**Table 3:** Concentration ranges and mean values (*n* = 6) of target contaminants in wastewater influent and limit of quantitation (LOQ) with standard deviation in parentheses (*n* = 6).

Compounds	LOQ (ng/L)	Range (μg /L)	Mean (μg /L)
Caffeine	10.6 (4.1)	8.53–33.7	18.4
Atenolol	8.54 (3.32)	0.012–0.409	0.166
Metoprolol	9.85 (4.56)	0.437–3.21	1.73
Amoxicillin	6.11 (4.07)	0.008–0.035	0.02
Trimethoprim	11.2 (7.35)	n.d. −0.023	0.007
Sulfadimidine	4.75 (2.32)	0.005–0.131	0.059
Sulfamethoxazole	6.83 (3.22)	0.012–0.092	0.037
Norfloxacin	10.63 (6.12)	0.014–0.226	0.106
Ofloxacin	14.46 (10.32)	0.1–0.912	0.560
Ciprofloxacin	3.65 (1.55)	n.d. −0.089	0.034
Lomefloxacin	2.12(1.46)	n.d. −0.0388	0.01
Enrofloxacin	3.56 (1.47)	n.d. −0.008	0.004
Oxytetracycline	2.66 (1.75)	0.009–0.035	0.018
Tetracycline	2.32 (1.02)	0.003–0.008	0.023
Chlortetracycline	4.11 (1.36)	n.d. −0.022	0.008
Doxycycline	2.33 (1.21)	n.d. −0.08	0.018
Clarithromycin	35.11 (15.88)	n.d. −1.26	0.368
Erythromycin-H_2_O	43.4 (45.9)	n.d. −0.082	0.020
Roxithromycin	35.3 (15.44)	n.d. −0.253	0.079
Azithromycin	23.12 (4.98)	0.047–4.42	1.41
Carbamazpine	3.13 (1.78)	n.d. −0.032	0.014
Benzhabeite	20.3 (8.65)	0.022–0.151	0.074
Bisphenol A	16.46 (6.21)	0.3–1.52	0.833
Nonylphenol	15.2 (10.6)	0.126–0.873	0.421
Estrone	7.65 (3.32)	0.078–0.158	0.106
17β-Estradiol	10.1 (4.40)	0.011–0.054	0.030
Estriol	8.32 (4.67)	0.042–0.162	0.092

n.d.: not detectable (below the LOQ).
